# Systematically Developing a Web-Based Tailored Intervention Promoting HPV-Vaccination Acceptability Among Mothers of Invited Girls Using Intervention Mapping

**DOI:** 10.3389/fpubh.2018.00226

**Published:** 2018-09-28

**Authors:** Mirjam Pot, Robert A. C. Ruiter, Theo W. G. M. Paulussen, Annerieke Heuvelink, Hester E. de Melker, Hans J. A. van Vliet, Hilde M. van Keulen

**Affiliations:** ^1^Child Health, Netherlands Organization for Applied Scientific Research (TNO), Leiden, Netherlands; ^2^Department of Work & Social Psychology, Maastricht University, Maastricht, Netherlands; ^3^Perceptual and Cognitive Systems, Netherlands Organization for Applied Scientific Research (TNO), Soesterberg, Netherlands; ^4^Centre for Infectious Disease Control, National Institute for Public Health and the Environment, Bilthoven, Netherlands

**Keywords:** HPV-vaccination acceptability, intervention mapping, eHealth, web-based intervention, design rationale

## Abstract

**Background:** Currently, the eHealth field calls for detailed descriptions of theory-based interventions in order to support improved design of such interventions. This article aims to provide a systematic description of the design rationale behind an interactive web-based tailored intervention promoting HPV-vaccination acceptability.

**Methods:** The 6-step Intervention Mapping (IM) protocol was used to describe the design rationale. After the needs assessment in Step 1, intervention objectives were formulated in Step 2. In Step 3, we translated theoretical methods into practical applications, which were integrated into a coherent intervention in Step 4. In Step 5, we anticipated future implementation and adoption, and finally, an evaluation plan was generated in Step 6.

**Results:** Walking through the various steps of IM resulted in a detailed description of the intervention. The needs assessment indicated HPV-vaccination uptake remaining lower than expected. Mothers play the most important role in decision-making about their daughter's immunization. However, they generally feel ambivalent after they made their decisions, and their decisions are based on rather unstable grounds. Therefore, intervention objectives were to improve HPV-vaccination uptake and informed decision-making, and to decrease decisional conflict among mothers of invited girls. Computer-tailoring was chosen as the main method; virtual assistants were chosen as a practical application to deliver interactive tailored feedback. To maximize compatibility with the needs of the target group, a user-centered design strategy by means of focus groups and online experiments was applied. In these, prototypes were tested and sequentially refined. Finally, efficacy, effectiveness, and acceptability of the intervention were tested in a randomized controlled trial. Results showed a significant positive effect of the intervention on informed decision-making, decisional conflict, and nearly all determinants of HPV-vaccination uptake (*P* < 0.001). Mothers evaluated the intervention as highly positive.

**Discussion:** Using IM led to an innovative effective intervention for promoting HPV-vaccination acceptability. The intervention maps will aid in interpreting the results of our evaluation studies. Moreover, it will ease the comparison of design rationales across interventions, and may provide leads for the development of other eHealth interventions. This paper adds to the plea for systematic reporting of design rationales constituting the process of developing interventions.

## Background

Too often design rationales of behavioral intervention programs are poorly described, leading to so-called “black box” evaluations (1). Currently, there is call in the eHealth field to open these black boxes. The scientific literature still provides detailed descriptions of *how* interventions are evaluated, but hardly ever of *what* exactly is being evaluated (2). Moreover, information about when and how decisions are made throughout the process of intervention development is often incomplete or even completely lacking (2, 3). Intervention development is a complex and laborious process which requires a large scale of decisions to be made along the way. This goes far beyond the decision about which behavior change techniques to include in an intervention (2, 3). We consider all of the decisions to represent valuable knowledge for the scientific community and for intervention developers who like detailed background about the conditions for (in)effectiveness of an intervention. Consequently, all decisions that were made during intervention development should be reported.

Hence, this article aims to provide a detailed, systematic description of the design rationale behind an interactive Web-based tailored intervention promoting HPV-vaccination acceptability. This paper encompasses all decisions that were made during the process of intervention development. A systematically developed and well described intervention enables the identification of active ingredients, improvement of existing interventions, future intervention development, and large-scale dissemination (1, 4). In addition, it facilitates comparison between interventions, for example for reviews and replication of studies (5–7). After all, the usefulness of systematic reviews depends on the quality of the studies included (3). Finally, it contributes to theory development by providing insight into causal mechanisms (3, 4, 7–9). We used the Intervention Mapping (IM) protocol, which provides a highly structured approach in describing an intervention program and its development (10).

## Methods

IM is a systematic process for developing theory- and evidence-based health promotion interventions. The IM protocol describes the pathways from problem identification to solution (10). The six steps of IM comprises several tasks, each of which integrates theory and evidence. The deliverable of completing the tasks within a step serves as a guide for the subsequent steps. Although IM is presented as a series of steps, Bartholomew Eldredge et al. (10) emphasize that the planning process is iterative instead of linear, meaning that intervention planners move back and forth between the various tasks and steps. By explicitly reporting all decisions and considerations throughout the intervention process, IM makes the intervention development process transparent.

**Figure 1:**
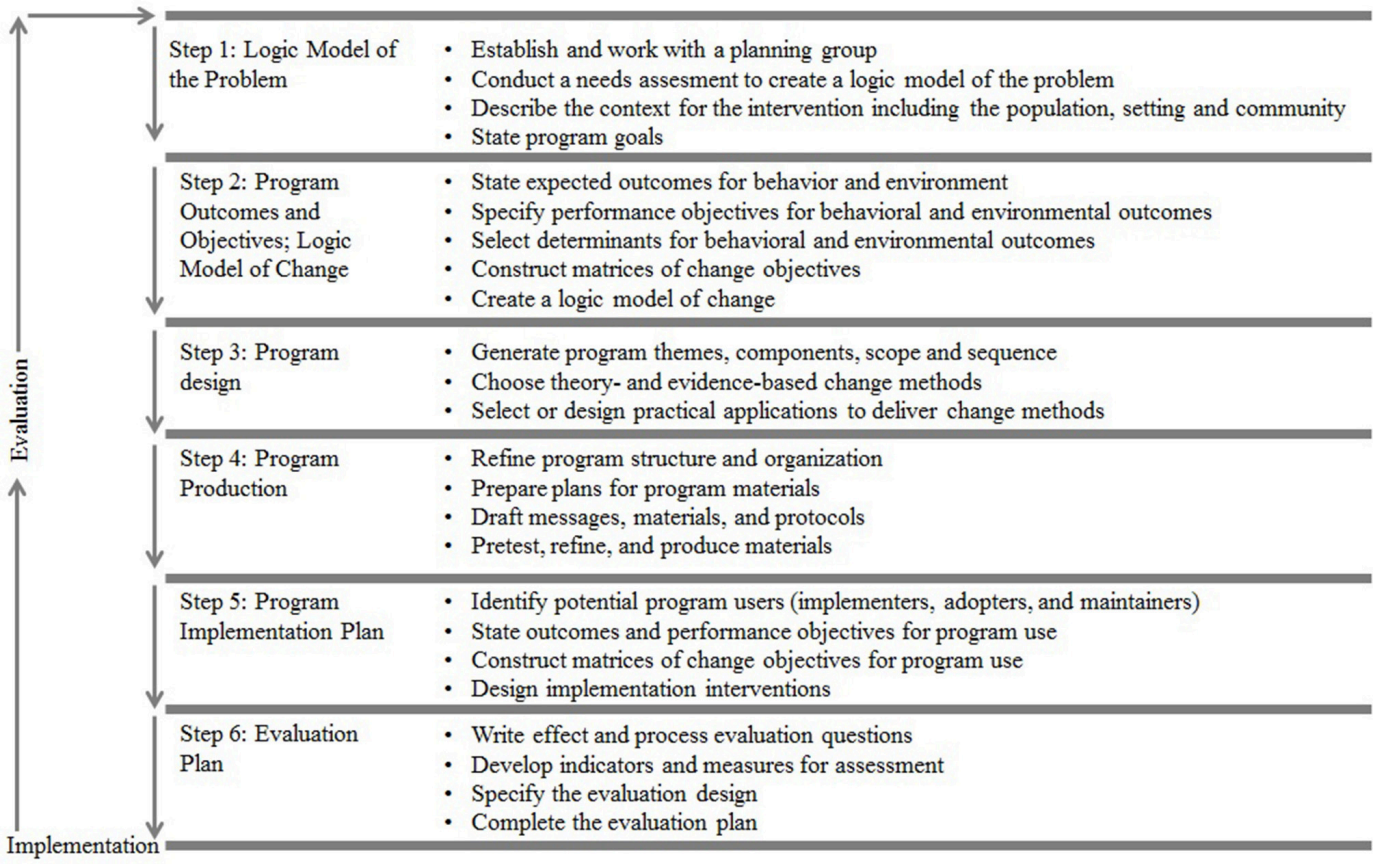
Intervention Mapping steps and tasks (adapted from Bartholomew Eldrigde and colleagues) (10).

Step 1 concerns the conduction of a needs assessment and formulation of the overall goals of the intervention. In this step, the health problem, behavioral, and environmental causes of this problem, and related determinants are identified. The intervention goal is the desired outcome of the intervention. In Step 2, performance objectives and change objectives are formulated. Performance objectives (POs) specify the (sub)behaviors that must be performed by the target group in order to reach the intended goal. Change objectives (COs) outline the specifics of behavioral determinants to be targeted so the target group is enabled to reach the performance objectives. COs are formed by crossing the POs with the determinants. This results in a matrix which can be seen as the core of the design rationale. Step 3 is about the design of the intervention program in terms of generating program themes, components, scope and sequence. The scope is the breadth and amount of the program and the sequence is the order in which programs are delivered across time. This step also includes the selection of theory-based intervention methods and the translation of these methods into practical applications, taking into account the parameters for effectiveness of the these methods. In Step 4, the methods and practical applications are being translated into a coherent intervention program In Step 5, adoption, implementation, and sustainability of the intervention in real-life settings are planned. Finally, Step 6 entails the outline of the process and effect evaluation. The steps and tasks of IM are visualized in Figure 1.

## Results

Systematically walking through all of the steps of IM, resulted in a detailed description of the intervention. This description, in turn, provides insight into the theory- and research-based foundations of the many decisions that were made during the process of intervention development. Below, the study findings from each step of IM are described.

### IM step 1: needs assessment

Worldwide, cervical cancer is the third most common cancer among women (11). Persistent infection by the human papillomavirus (HPV) appears to be the major cause of cervical cancer (12). In the Netherlands, yearly 600 new cases of cervical cancer are detected, of which 200 with fatal consequences (13). This is despite the presence of a national cervical cancer screening program for women aged 30–60 years (14). HPV-vaccinations of 12-year-old girls were expected to reduce the number of cervical cancer cases by 50% (13). Therefore, in 2008, the Dutch government decided to include the HPV-vaccination of 12-year-old girls in the National Immunization Program (NIP). However, HPV-vaccine uptake remains lower (53%) than expected (70%) (15). There is a need to improve this uptake is order to reduce the cervical cancer burden. Therefore, the primary goal of the intervention was to improve HPV-vaccination uptake among invited girls.

Most studies have confirmed that parents play a large role in decision-making about their daughters' HPV-vaccination [e.g., (16–18)]. A Dutch study showed mothers to play the most important role in the immunization decision of girls. There is a high percentage of consensus between mothers and daughters (79%) about the outcome (19). Therefore, mothers were selected as the target group for designing an educational intervention for promoting the HPV-vaccination uptake by their daughters In order to gain insight into determinants of mothers' HPV vaccination acceptability, we conducted a longitudinal study (20). The results showed that intention was the main and stable predictor of HPV vaccination uptake. Intention, in turn, was best explained by attitude, behavioral beliefs, subjective norms, habit, and perceptions about the relative effectiveness of the vaccine, they explained 83% of the variance in HPV-vaccination intention. Also relevant for the mothers' intention were anticipated regret, risk perception, self-efficacy, and knowledge. Additional determinants of HPV-vaccination acceptability found by previous cross-sectional studies were confidence in authorities, ambivalence, and processing of HPV-vaccination education (19, 21).

Furthermore, research showed that a substantial proportion of the mothers had not actively processed information about the HPV-vaccination (50%) and still felt ambivalent after they made their decision (25%) (19). This indicates that the mothers' decision is based on rather unstable grounds, which makes them vulnerable for arguments challenging their initial attitudes and/or intention. Informed decision-making is expected to make mothers less vulnerable for counter arguments (22, 23). Furthermore, decisional conflict is strongly related to informed decision-making, as one of the factors contributing to decisional conflict is feeling uninformed (24). A more informed decision is thus theoretically related to reduced decisional conflict (25). Hence, the secondary goal of the intervention is to improve informed decision-making, reduce decisional conflict, and positively influence determinants of HPV-vaccination uptake.

In the Netherlands, the existing education about the HPV-vaccination consists of an introduction folder and a link to a website providing generic information. All girls at the age of 12 receive an invitation for the HPV-vaccination with the accompanying brochure and link to the website. But, this education needs to be improved because HPV-vaccination uptake remains lower than expected (15). Besides, Van Keulen et al. (19) showed that mothers indicated a need for more interactive, personal information about the HPV-vaccination over and above the general information. Mothers also expressed differential needs concerning the amount and scope of information. Topics of interest for future communication about the HPV-vaccination as indicated by mothers were for example, the pros and cons, potential long-term side effects, and the safety and effectiveness of the HPV-vaccination (19). Regarding the delivery mode of the information, mothers indicated a preference for internet (19). These preferences guided our decision to develop a web-based intervention, enabling us to provide mothers with interactive, tailored information about the HPV-vaccination (see Step 3).

### IM step 2: program outcomes and objectives

#### Program outcomes

Based on the identified problem and needs we determined that the primary outcome was to improve HPV-vaccination uptake among invited girls and the secondary outcome was to strengthen mothers' informed decision-making, reduce decisional conflict, and positively influence determinants of the HPV-vaccination decision. Below, we introduce POs, determinants of these POs, and accompanying COs for each outcome.

#### Performance objectives

The expected intervention outcomes were subdivided into POs. The HPV-vaccination consists of two subsequent injections. In other words, the behavior has to be repeated only once (with an interval of 6 months). The POs are: (1) the mother makes a (informed) decision to have her daughter vaccinated against HPV; (2) the mother discusses her decision with her daughter and partner; (3) the mother guides her daughter toward receiving the first HPV-injection; (4) the mother guides her daughter toward receiving the second HPV-injection.

#### Behavioral determinants

For each PO, we identified the reasons why mothers would take that action. These so called behavioral determinants were based on theory (e.g., the theory of reasoned action and socio-cognitive theory) (25, 26) and on empirical research (19, 20, 27). We selected determinants that met the criteria of importance and changeability (10). Importance of the determinants of PO 1 (i.e., deciding to get the HPV-vaccination) was based on the association (i.e., R2 effect size) (28) of the determinants with HPV-vaccination intention (21). For the POs 2–4 (i.e., discussing the HPV-vaccination and actually getting the first and second injection), importance was based on consensus among co-authors (RR, TP, MP, and HvK). Changeability (i.e., the strength of the evidence that the proposed change can be realized by the intervention), was also based on consensus among co-authors (RR, TP, MP, and HvK).

For the first PO, we returned to the needs assessment and selected the following determinants: attitude, beliefs, positive, and negative outcome expectancies, anticipated regret about both receiving and rejecting the HPV-vaccination, confidence in authorities, habit strength, risk perception having received (no) HPV-vaccination, subjective, and descriptive norms, relative effectiveness of the HPV-vaccination, ambivalence, and HPV-vaccination information processing (19, 21). Furthermore, according to Marteau et al. (27), an informed decision is based on sufficient and relevant knowledge, and a match between the person's values (i.e., their attitude toward the HPV-vaccination) and outcome behavior (i.e., whether mothers had their daughter vaccinated against HPV or not). Consequently, knowledge was selected as a determinant. Determinants that were selected for PO2 were attitude (29), and self-efficacy (26). Selected determinants for PO3 and PO4 were knowledge and beliefs (26). Determinants that were not selected were considered either unimportant (e.g., for PO1: self-efficacy) (19, 21) or unchangeable (e.g., for PO2: parenting style).

#### Change objectives

For each determinant, we identified COs. COs are the active ingredients of the intervention and function as a blueprint of the theoretical design rationale. Table 1 provides an overview of examples of the matrix of COs, the complete version can be found in Additional File 1.

**Table 1:** Examples of change objectives (COs).

**Performance objective**	**Determinant**			
	**Knowledge**	**Attitude**	**Beliefs**	**Risk perception having received no HPV-vaccination**
1. Mother makes the (informed) decision to have her daughter vaccinated against HPV.	Mother explains that HPV is a virus. Mother explains that HPV is transmitted sexually. Mother explains that men can also be infected with HPV.	Mother evaluates the HPV-vaccination positively. Mother recognizes the health benefits of the HPV-vaccination.	Mother recognizes the importance of her daughter receiving the HPV-vaccination before they become sexually active (i.e., age 12). Mother recognizes that the vaccine has proven to be safe and effective.	Mother acknowledges the risk of her daughter becoming infected with HPV and developing cervical cancer later in life without the vaccination.
2. Mother discusses her decision to have her daughter vaccinated against HPV with her daughter and partner.		Mother evaluates communication with her daughter and partner positively.		
3. Mother guides her daughter toward receiving the first HPV-injection.	Mother knows where to get the first HPV-injection.			
4. Mother guides her daughter toward receiving the second HPV-injection.	Mother knows where to get the second HPV-injection.		Mother recognizes that the HPV-vaccination is most effective when her daughter gets fully vaccinated.	

### IM step 3: program design

#### Theme, components, scope and sequence

The first task of Step 3 is to generate ideas for intervention theme, components, scope, and sequence. The product of this step is an initial plan that describes the program (10). We decided the intervention to be Web-based (see Step 1). The main theme of the intervention was “making an informed decision about the HPV-vaccination of your daughter.” The various components were logically clustered for improving usability. This resulted in four menu options: (1) two-sided information about the HPV-vaccination, (2) a decisional balance, (3) practical information, and (4) frequently asked questions (See Additional File 3 for screenshots of the four menus). The first menu enabled mothers to collect tailored information about the HPV-vaccination (e.g., such as information about the effectiveness of the HPV-vaccination). The various components were in line with mothers' preferences as indicated by earlier research (See “needs assessment”) (10). In the second menu, mothers could weigh their personal values regarding the HPV-vaccination in the form of a decisional balance and values clarification tool. In the third menu, mothers could gather practical information such as how and where to receive the HPV-vaccination. The fourth menu listed frequently asked questions about the HPV-vaccination. Here we also added a “problems with the website” component, providing mothers with help.

Furthermore, mothers were able to visit the intervention multiple times. The first time they visited the website, they were provided with an explanation of how the website worked. Then, they were introduced to the first menu. We used a combination of a freedom of choice and a tunneled design (i.e., a “hybrid design”) to guide mothers through the website (30). This means that mothers could choose themselves which component in which menu they wanted to visit (i.e., freedom of choice design). However, once they entered a component, they were guided through it in “tunnel fashion,” with navigation being limited to “next” and “prior” buttons. The reason for choosing such a hybrid design is that we wanted to profit from the strengths of both approaches (30). Specifically, the tunnel design was expected to increase intervention adherence and engagement and acquisition of knowledge (31). The freedom of choice design was expected to promote mothers' autonomy, which is important when motivating behavior change (32, 33). Furthermore, the hybrid design matched the differential needs concerning the amount and scope of information expressed by the mothers (19). Table 2 provides an overview of the scope and sequence of the intervention.

**Table 2:** Scope (components and main targeted determinants) and sequence of the intervention.

**Menu^a^**	**Component^b^: main targeted determinants**
Information about the HPV-vaccination	General information: *knowledge*
	Facts and stories: *beliefs, positive and negative outcome expectancies*
	From HPV to cervical cancer: *knowledge*
	Ways to protect against cervical cancer: *relative effectiveness*
	Side effects of the HPV-vaccination: *negative outcome expectancies*
	Importance vaccinating at young age: *positive outcome expectancies*
	Other mothers: *descriptive norm*
	Working mechanisms vaccination: *knowledge*
	Chance of getting HPV/cervical cancer: *risk perception having received (no) HPV-vaccination*
	Effectiveness and safety of the HPV-vaccination: *beliefs, positive outcome expectancies*
Weighing up the pros and cons	Decisional Balance: *attitude, ambivalence*
	Values clarification: *attitude, ambivalence*
Practical information	Talking about the HPV-vaccination: *attitude, self-efficacy, subjective norms*
	Where do I get the HPV-vaccination: *knowledge, planning, self-efficacy*
	2 instead of 3 HPV-injections: *knowledge*
Frequently asked questions	Frequently asked questions about the HPV-vaccination: n/a
	Frequently asked questions about getting the HPV-vaccination: n/a
	Problems with the website: n/a

*n/a, not applicable*.

a *Within and across the different menus, a freedom of choice design was used*.

b *Within the various components, a tunnel design was used*.

#### Theoretical methods and practical applications

To identify theoretical change methods that help achieve the COs, we used an overview of methods provided by Bartholomew et al. (chapter 6) (10). The eHealth setting gave us the chance to apply effective strategies in an innovative way, namely by using computer-tailoring and interactions with virtual assistants (see sections below) (34). Computer-tailoring was selected as the main theoretical framework for development. Tailoring is a health communication strategy by which messages are individualized to personal preferences and needs (35). Meta-analyses have shown that tailored interventions are more effective than generic interventions in achieving behavioral outcomes [e.g., (36, 37)]. Beneficial effects of tailoring are attributed to improved exposure, information processing, appreciation, reading, and perceived personal relevance [e.g., (38, 39)]. Because computer-tailored interventions can reach large groups of people at relatively low costs, especially when delivered via the Internet, (40), they can have substantial impact at the population level (41). Also, tailoring matches the mothers' need for more interactive, personal information about the HPV-vaccination (Step 1) (19).

Computer-tailored feedback was used in three different ways throughout the intervention. First, it was used to tailor the feedback on participants' answers to statements and questions about specific aspects of the HPV-vaccination. For instance, mothers were first asked to estimate their daughters' chance to get an HPV-infection. Those who perceived this chance as low, received feedback which stated that this chance is rather high instead of low, whereas those who perceived the chance as high, received feedback that confirmed that the chance is indeed high. Second, computer tailoring was used to provide mothers the opportunity to weigh their personal values regarding the HPV-vaccination in a decisional balance. Another mean was the “value clarification” tool [a motivational interviewing strategy; cf. (32)]. Mothers were invited to list their central values for life, and were stimulated to relate these to the decision about vaccinating her daughter. Finally, computer-tailoring was used for guiding mothers through the website. The intervention kept track of the components that the mother had already visited by using logs. This enabled us, for instance, to highlight parts of the intervention which the mother had not seen yet. Also, if mothers were exposed to information that had already been discussed in another component, the intervention mentioned this in order to assure that the connection between the different types of information provided was clear.

We selected virtual assistants for delivering tailored feedback. A virtual assistant is an embodied conversational agent defined as a computer program with a human-like visual make-up and appearance on a computer screen (42). Virtual assistants were chosen to match the mothers' preferences for more interactive personalized feedback (19). They provide opportunities for two-way interactions, and can create a highly personal experience. Also, research has indicated that a social relationship between user and program is important (43, 44), as it supports the basic psychological need for relatedness (33, 45). This can be established by using virtual assistants (46–48). Also, several studies confirmed that the presence of a virtual assistant can further improve the effectiveness of the intervention (49–51). Specifically, the added value of using a virtual assistant over a text and picture-based website is that it improves recall of information (52), transfer of learning (53), amount of learning (54), self-efficacy expectations, literacy, and behavior change (49, 50, 55). In addition, the mere presence of such an animated interface agent has a positive effect on experiencing fun and engagement [e.g., (47, 50, 56)]. Two virtual assistants were visualized: a mother-like and a female doctor-like assistant as the combination of using an expert and a peer virtual assistant has been shown to be effective (57, 58). The main purpose of the virtual assistants was to provide mothers with social support, which is an important factor associated with positive health outcomes in general (59). The mother-like assistant was used to guide mothers throughout the website and helped weigh their personal values in the decisional balance. The doctor-like assistant was used to deliver feedback about the HPV-vaccination.

Table 3 provides examples of theoretical methods (column 2) for determinants identified in IM step 2 (column 1) for PO1 (i.e., mother makes the informed decision to have her daughter vaccinated against HPV). For each method, parameters for effectiveness were specified (column 3). We then translated theoretical methods into practical applications (column 4) that were appropriate for the population and the (Internet) setting. In Additional file 2, we also specify which POs and COs were targeted using which methods and applications in the various components. This can be seen as the most straightforward blueprint of the intervention. All COs were covered by the intervention.

**Table 3:** Examples of selected methods, strategies, parameters and strategies for Performance Objective 1 “mother makes the (informed) decision to have her daughter vaccinated against HPV.”

**Determinants**	**Theoretical method**	**Parameter for use**	**Practical application**
Beliefs, positive and negative outcome expectancies	Belief selection (TRA^a^) Active learning (ELM^b)^	Requires investigation of the current attitudinal, normative and efficacy beliefs of the individual before choosing the beliefs on which to intervene Requires time, information and skills	“Facts & Stories”: mother is asked by the mother-like assistant to indicate for various statements, whether they are either a “fact” (true) or a “story” (false).Then, the doctor-like virtual assistant elaborates on correct outcome expectancies, beliefs, misperceptions and omissions.
Attitude, ambivalence	Decisional Balance (MI^c^)	Requires consideration and evaluation of behavior	“Weigh up the pros against the cons”: Mothers are presented with a list of pros and cons of the HPV-vaccination by the mother-like assistant. Based on pros and cons mothers marked as most salient, a decisional balance reveals their current position on a scale ranging between wanting and not-wanting to get my daughter vaccinated.
Attitude, ambivalence	Value Clarification (MI^c^) Modeling (SCT^d^)	Requires consideration and evaluation of values Attention, resemblance, self-efficacy and skills, reinforcement of the model, identification with the model, coping model instead of mastery model.	“What are your values?”: Mothers are invited to list their central values for life. Optional, they can find examples of values of other mothers (e.g., being a good parent). They will then be stimulated to relate these to the HPV-vaccination. Here, examples of how these values were related to the HPV-vaccination according to other mothers, were available.
Risk perception having received (no) HPV-vaccination	Statistical risk information (HBM^e^) Consciousness raising (HBM^e^) Framing (PMT^f^)	Can use feedback and confrontation; however, raising awareness must be quickly followed by increase in problem-solving ability and self-efficacy. Requires high self-efficacy expectations. Gain frames are more readily accepted and prevent defensive reactions	Mother-like assistant asks about mothers' perceived risk perception of her daughter getting infected with HPV and of her daughter developing cervical cancer. Tailored feedback on this perceived risk is then given by the doctor-like assistant. Finally, mothers are provided with statistical risk information (i.e., the probability rates of attracting HPV and cervical cancer).

a *TRA, theory of reasoned action (29)*.

b *ELM, elaboration likelihood model (60)*.

c *MI, motivational interviewing (32)*.

d *SCT, social cognitive theory (26)*.

e *HBM, health belief model (61)*.

f *PMT, protection motivation theory (62)*.

**Figure 2:**
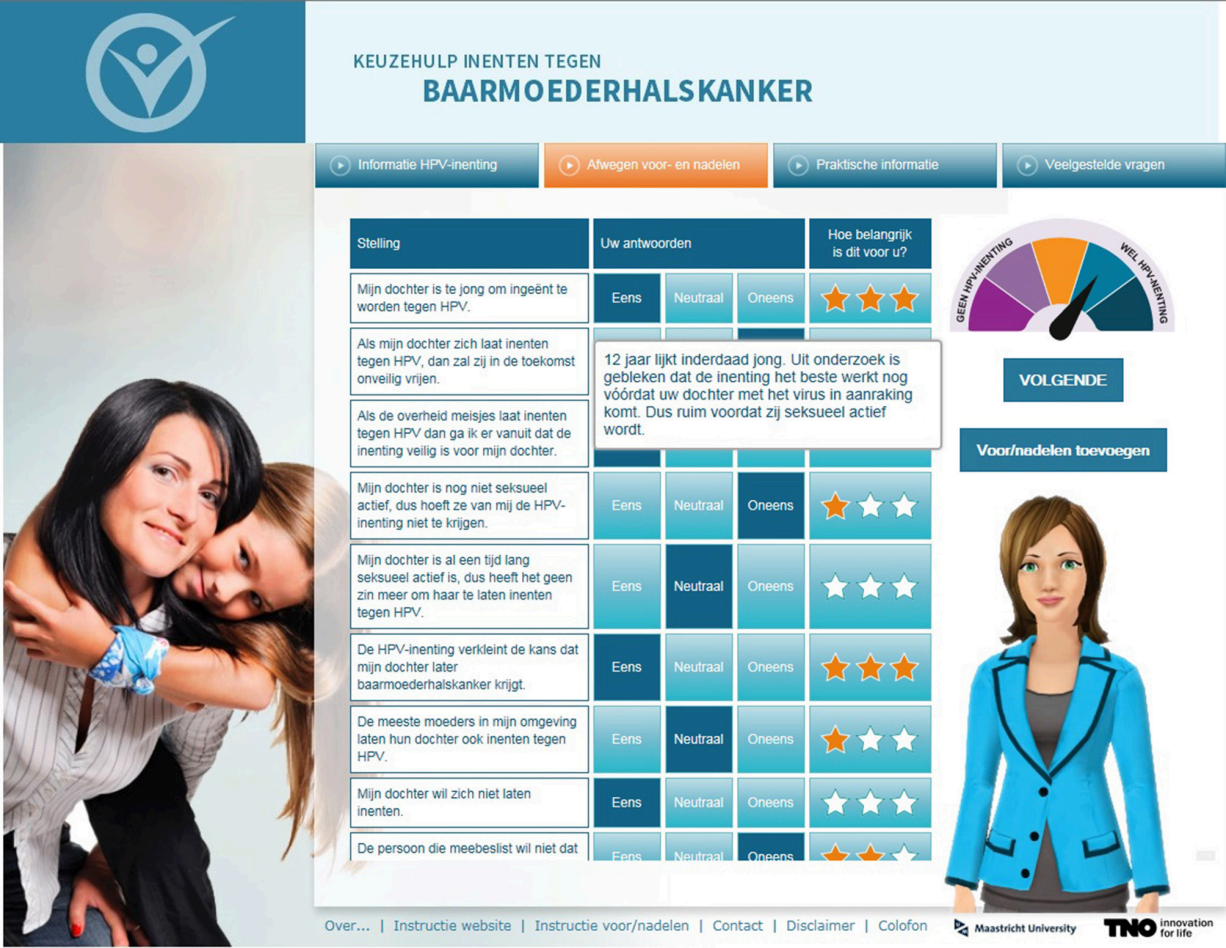
Screenshot of the decisional balance with a tailored pop-up and the mother-like virtual assistant on the website. *The plan for the decisional balance was developed in step 3; actual development of the balance was realized in step 4.

The most important method aiming to reduce decisional conflict was the decisional balance (see Figure 2), which has proved a quick and efficient intervention by itself (63). Mothers were presented with a list of pros and cons of the HPV-vaccination by the mother-like assistant (left column). This list was based on pros (e.g., “the HPV-vaccination decreases the chance of my daughter getting cervical cancer”) and cons (e.g., “my daughter is too young to receive the HPV-vaccination”) that were considered most important to the mothers as indicated by the needs assessment (Step 1). For each pro or con, they indicated (1) whether they agreed (disagree/neutral/agree; middle column) and (2) how important the pro or con was to them (unimportant/neutral/important; third column). The latter was indicated by stars: the more stars, the more important the pro or con was to the mother. When mothers (dis)agreed, tailored feedback “popped up.” This was done to ensure mothers based their answer on correct information (see Figure 2). Furthermore, mothers were given the option to add pros and cons that were not in the list. Based on pros and cons mothers marked as most salient, a decisional balance (top right of the screen) revealed their current position on a scale ranging between not-wanting (left side) and wanting (right sight) to get their daughter vaccinated.

### IM step 4: program production

**Figure 3:**
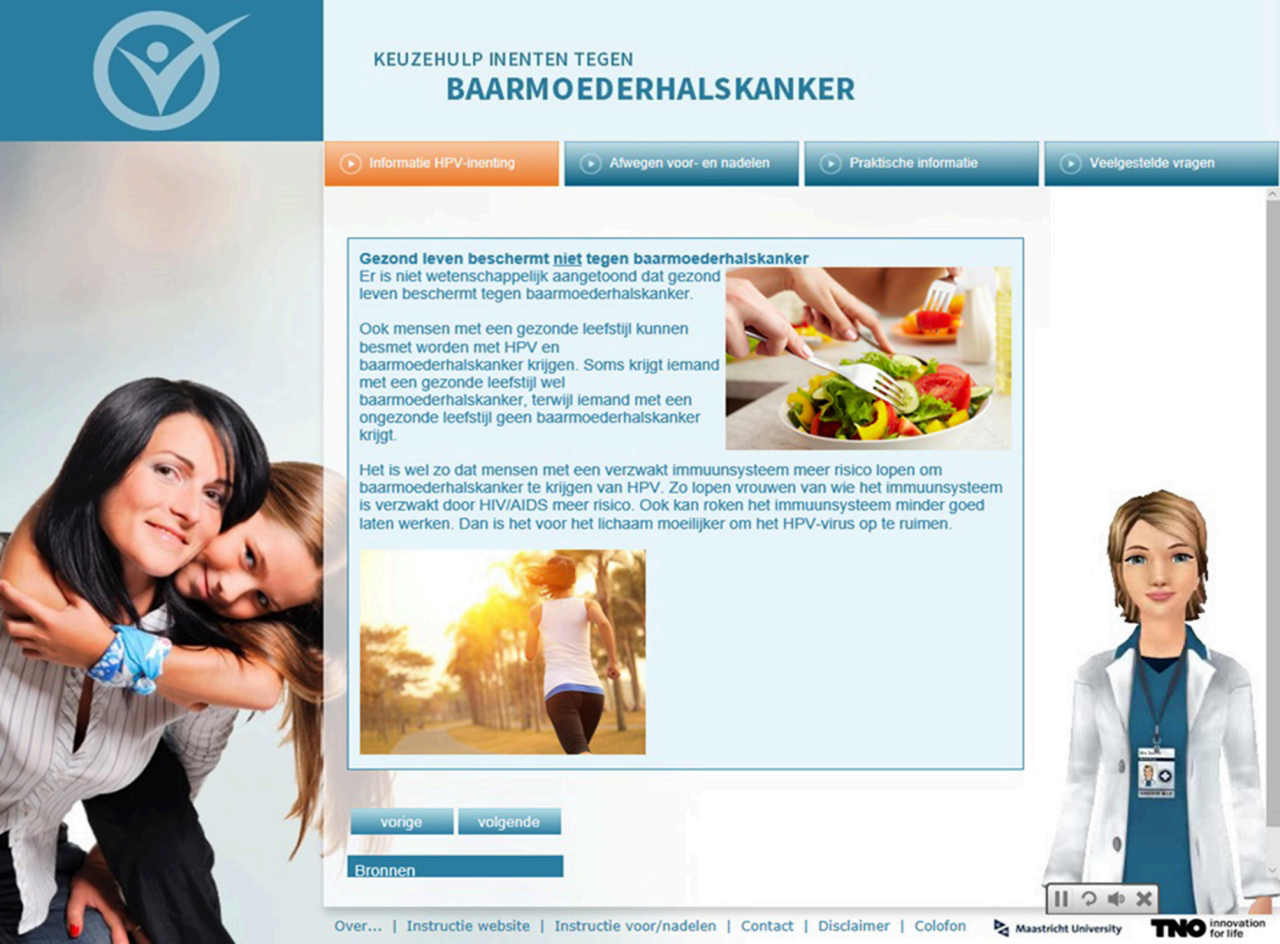
Screenshot of the doctor-like assistant providing feedback about the ineffectiveness of having a healthy life style (e.g., healthy eating, exercising) in protecting against cervical cancer with illustrations provided by a graphic designer.

We developed the intervention using Tailorbuilder^©^ software. The virtual assistants were developed by a company called “Webspeaking.” Individual responses and routing were linked to written and spoken feedback messages by means of computer software using if-then algorithms. The website was made available on computers and tablets and was OS-platform independent. Using an online questionnaire, mothers (*N* = 375) were asked about the preferred graphical appearance of the intervention (including the name of the intervention, the voices and appearances of the virtual assistants). A text-editor rephrased the written and spoken texts in order to maximize comprehensibility. A graphic designer made the website design and provided us with appropriate pictures to illustrate feedback, in order to make the website more appealing for this target group (See Figure 3 for an example).

#### Experimental pre-testing and pilot-testing of prototypes

In order to anticipate an intervention that meets the requirements and preferences of the target group, we followed user-centered design procedures. This entails the iterative involvement of the end-users in the design process (64). We gathered feedback on different versions (static and interactive) or (parts of the) intervention, by online experimental pretests and focus groups, respectively. Experimental pretesting offers empirical support for the impact of strategies on determinants and serves as a guarantee for implementing adequate intervention materials (65, 66). We conducted three experimental online pretests. Since we did not find a clear consensus in the literature about the framing of risks, the first experiment (*N* = 375) was about testing the differential effect of providing statistical (i.e., probability rates) or/and narrative risk information (i.e., a personal story). Mothers were randomly assigned to one of four conditions in a 2 (statistical information: yes or no) × 2 (narrative information: yes or no) between-subjects factorial design. ANOVA revealed a significant main effect of statistical information on daughters' perceived susceptibility toward HPV [F_(1, 371)_ = 7.56, *p* < 0.01]. Mothers who received statistical risk information had a higher perceived daughters' susceptibility toward HPV (M = 4.11 on a 7-point scale, SD = 0.10) than mothers who did not receive statistical risk information (M = 3.73 on a 7-point scale, SD = 0.09). Thus, statistical risk information seemed most effective. We therefore decided to include statistical risk information to target risk perception with this intervention (Step 3).

In a second online experimental pretest (*N* = 561), we explored the best way to communicate about social norms; by providing negatively (i.e., discourage undesired behavior) vs. positively (i.e., encourage desired behavior) framed descriptive and/or subjective norms (67). It was suggested that the descriptive norm should be avoided in situations where the unhealthy behavior is prevalent (68). As for the HPV-vaccination, 39% of invited girls have *not* received the HPV-vaccination (15). Therefore, we also wanted to examine whether communicating a descriptive norm could have a potential adverse effect on HPV-vaccination acceptability. Mothers were randomized into one of four conditions in a 2 (norm: injunctive vs. descriptive) × 2 (frame: positive vs. negative) between subjects factorial design with an additional control condition. We found no indication for using one type of framing norms over the other (*p*'s > 0.05; mean HPV-vaccination intention scores ranging from 5.51 to 5.77, on a 7-point scale). We also did not find any adverse effects of descriptive norms on HPV-vaccination acceptability (*p* > 0.05). As negatively framed norms were expected to be more difficult to process (69), we decided to just include positively framed descriptive norm by communicating about the national HPV-vaccination uptake rate. Within the component “talking about the HPV-vaccination,” mothers were taught how to deal with a potential contrasting subjective norm of important others (e.g., their daughter and partner).

Finally, being a relatively new vaccine, there remains uncertainty about potential long-term effects of the HPV-vaccination. This was also found to be a topic of interest among mothers for future communication (19). Therefore, in a third experimental pretest (*N* = 695), we investigated the effects of acknowledging vs. ignoring uncertainty about potential long-term effects of the HPV-vaccination. Mothers were randomly assigned to one of two conditions in which uncertainty about the HPV-vaccination was either (a) acknowledged or (b) ignored. Results showed that, compared to mothers who were exposed to information ignoring uncertainty, mothers who were exposed to information acknowledging uncertainty experienced more decisional conflict (acknowledged: M = 3.42, SD = 1.84, *p* < 0.01 vs. ignored: M = 3.05, SD = 1.74), were more ambivalent about their decision (acknowledged: M = 4.04, SD = 1.86 vs. ignored: M = 3.42, SD = 1.89, *p* < 0.001), and had a less positive attitude (acknowledged: M = 5.07, SD = 1.50 vs. ignored: M = 5.69, SD = 1.38, *p* < 0.01) and intention (acknowledged: M = 5.26, SD = 1.73 vs. ignored: M = 5.85, SD = 1.45, *p* < 0.01). These findings implicate *not* to communicate about long-term uncertainties. However, we chose to do so in the intervention, for the following reasons: first, the found effect sizes were small. Second, not communicating about long term uncertainties brings along the risk of mothers searching information about this elsewhere. This can be quite dangerous as many rumors about potential long-term effects, for which no prove exists, can be found (e.g., on the Internet) (70). Reading these (false) rumors without any refutation being offered aside (71), could have more detrimental effects on HPV-vaccination acceptability than when we ourselves provide the (correct) information. The latter enables us to inoculate mothers with arguments that become accessible in case they are confronted with (new) information that might challenge their initial positive intentions (i.e., psychological inoculation) (22, 23). Finally, mothers themselves expressed a need for full disclosure, especially when uncertainties were ignored, which was also found in a previous study (19).

At a later stage, we conducted several focus groups (*N* = 3) among mothers to test interactive prototypes of the intervention to ensure compatibility with the preferences of the target group. A first prototype of the intervention was tested in two focus groups. After we revised the prototype according to the feedback from these two focus groups, a second prototype of the intervention was tested in a third focus group. The protocol was similar for all focus groups: after a general introduction, mothers were given a laptop and headset to individually navigate through the website. They were given the opportunity to give feedback on every page of the website about features they (dis)liked (e.g., the “look and feel” of the page(s), and the tailored feedback of the virtual assistants). Then, they were asked to fill out a written questionnaire assessing their subjective evaluation of the virtual assistants (e.g., the extent to which feedback matched their responses) and the website (e.g., their evaluation of the different menus). Finally, in a group discussion mothers could elaborate on their opinion about the intervention, and offer suggestions for improvement.

Feedback was first gathered from the first two focus groups. For instance, in the first prototype, there was a component targeting anticipated regret by using imagery (72, 73). Mothers were asked how much regret they would have if they did not vaccinate their daughter against HPV and their daughter developed cervical cancer later in life. However, we discovered that asking this evoked much resistance. We therefore decided to remove this component from the intervention. As an alternative, we decided to target anticipated regret indirectly (e.g., by giving information about the high prevalence of HPV). Furthermore, in the first prototype, the written and spoken tailored feedback were provided at the same time. Mothers indicated that, therefore, they experienced difficulties listening to the virtual assistant. Hence, we created a new prototype, in which the written feedback appeared once the virtual assistant was done providing the tailored feedback.

**Figure 4:**
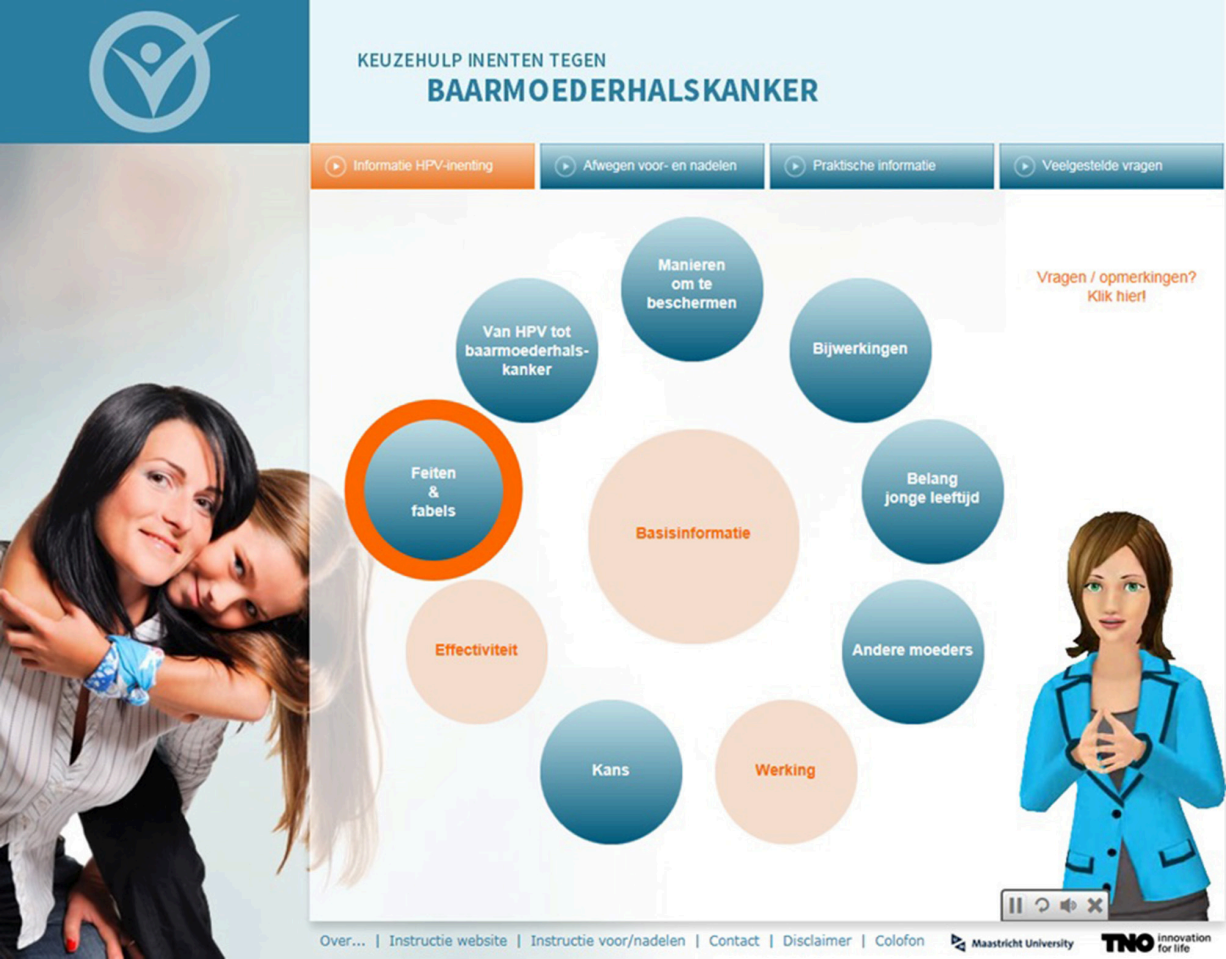
Screenshot of the first menu with the mother-like assistant in which a suggested component is highlighted and visited components have turned into a different color.

In the third focus group, mothers indicated that they would like to see which components they had already visited. We therefore created an adapted version of the website in which logs were used to register the pages mothers had already visited and subsequently used these logs to visualize which components were completed. This was done by turning them into a different color (i.e., orange, see Figure 4) In addition, the mother-like virtual assistant was used to give advice about components to visit next, in order to maximize exposure to the intervention. If the virtual assistant advised on a component, the component was highlighted by an orange circle (see Figure 4).

After revising the intervention according to the feedback from the third focus group, a final prototype was pilot-tested online using various devices to ensure it worked adequately. This was done among a sample of mothers (*N* = 10) and among members of the project group.

### IM step 5: designing an implementation plan

To ensure future implementation and adoption of the intervention (step 5), we formed an advisory board of representatives of important linking agents (e.g., Public Health Services) and professionals involved in delivering the HPV-vaccination. We organized two advisory board meetings; they advised on the experimental pretesting, practicability, and feasibility of the intervention, the planned effect- and process evaluation, and implementation of the intervention within the NIP. The National Institute for Public Health and the Environment (RIVM), responsible for the national implementation of HPV-vaccination, was co-financier of the project and full member of the project team. RIVM would get full control and management over the website if the final intervention turned out to be effective.

### IM step 6: creating an evaluation plan

In order to evaluate the efficacy and effectiveness of the intervention, we planned a randomized controlled trial (RCT). The RCT consisted of 2 arms: (1) a control and (2) intervention group. Mothers were randomly recruited from Praeventis, the Dutch National Immunization Register, and three Internet panels. The latter was to guarantee a suitable subsample for the planned efficacy trial (21The Praeventis sample enabled us to anticipate the naturalistic condition for future implementation of the intervention, which provided the opportunity for testing the intervention's effectiveness. The primary outcome measure was HPV-vaccination uptake, as registered by Praeventis Secondary measures were informed decision-making, decisional conflict, and determinants of HPV-vaccination acceptability. These were measured using a Web-based questionnaire.

Part of the RCT was a process evaluation assessed program adherence and the users' subjective program evaluation. At follow up, participants evaluated the information provided by the website (e.g., relevance, credibility), perceived user control (e.g., experienced degree of autonomy) and the functioning of the virtual assistants (e.g., fun, reliability). Mothers were also asked to rate the website and the virtual assistants on a 10-point scale, ranging from 0 (very bad) to 10 (excellent). Objective program use was evaluated by the logs keeping track of the pages the mothers' has visited. Two indicators were computed: “completeness” and “time.” Completeness represents the total percentage of pages that a participant has visited while logged into the website, ranging from 0% (no exposure) to 100% (exposure to all pages). Time represents the total amount of time participants have spent logged into the intervention.

Results from the RCT are described in detail elsewhere (74). The main finding from the effect evaluation was that the intervention showed a significant positive effect on informed decision-making, decisional conflict, and nearly all determinants of HPV-vaccination uptake (*P* < 0.001). No differences in intervention effects were found between the two differential samples. The main finding from the process evaluation was that mothers evaluated the intervention as highly positive: mothers evaluated the website with a 7.6 (SD = 1.36) and the virtual assistants with a 7.4 (SD = 1.53). According to the computer logs, 2,509 (63%) of the 3,995 (100%) invited mothers logged on to the website. On average, mothers spent 22 minutes on the website (SD = 13 min).

## Discussion

In this article, we have provided a comprehensive and detailed description of how we systematically developed an intervention promoting HPV-vaccination acceptability using the IM protocol. This led to a highly innovative, interactive, Web-based, tailored intervention, in which tailored feedback was delivered by virtual assistants. Tailoring has only recently been applied to HPV-vaccination (57, 75–77). To our knowledge, only one of the existing tailored interventions was computer-tailored and this intervention turned out to be ineffective in promoting HPV-vaccination acceptance (75). Moreover, not only did we tailor the content of the intervention to the mothers' personal interest, but tailoring was also used to guide the mothers' personal pathway through the intervention. The latter is likely to have improved the usability of the intervention. The intervention accounted for tailoring on a variety of determinants. For example, not only did we tailor on perceived barriers (e.g., beliefs about adverse effects), like Gerend et al. (76) did, but also on other beliefs (e.g., beliefs about the daughters' sexual behavior and age in relation to the need for the HPV-vaccination), attitude, subjective norms, habit, relative effectiveness, anticipated regret, risk perception, self-efficacy, and knowledge. The use of virtual assistants in interventions promoting HPV-vaccination acceptance seems promising since results from the focus groups (Step 4) and the subjective program evaluation (74) showed that mothers appreciated them very well. But, we still consider the use of virtual assistants to be complex, especially in Web-based interventions in which both spoken and written feedback/information are provided.

The intervention appeared effective in promoting HPV-vaccination acceptability and informed decision-making, and appeared to have potential for broad scale dissemination and implementation (74). This intervention blueprint will aid in interpreting the results of our evaluation studies (74). In addition, it provides insight into causal mechanisms, which contributes to theory development (3, 4, 7–9). Moreover, it will ease the comparison of design rationales across interventions (e.g., for reviews and replication of studies) (5–7). Finally, it provides leads for the development of other eHealth interventions (1, 4).

### Advantages of IM

We believe that using IM greatly contributed to the intervention being effective in promoting HPV-vaccination acceptability and informed decision-making among mothers of invited girls. First, by developing the intervention in a systematic manner, we ensured a solid theoretical and empirical foundation for the intervention [cf. (10)]. For decisions to be made about methods/applications that lack a solid ground of consensus in the research literature, we were able to pre-test the impact of alternative prototypes before finalizing and testing the full operational intervention.

Furthermore, according to IM, it is imperative that members of the target group are involved in the development of the intervention (10). However, currently, in many eHealth interventions, the design of the intervention is based on assumptions that are not validated with input from end-users. In fact, the importance of formative research and pretesting of materials is often being overlooked. The resulting intervention may therefore lack key features, and subsequent evaluations of the effectiveness of the interventions may be compromised (78). Therefore, we applied a user-centered design by extensively involving mothers in the intervention development from the beginning to the end (64). This was done by conducting focus groups and online experimental pretests, in which we gathered feedback from representatives of the target group. This iterative process of development and feedback guided our attempt to gradually improve the solution we had to offer for reaching the intended objectives. In other words, erroneous or inconclusive decisions can thoughtfully be changed or reversed in order to prevent the final intervention from being at odds with the objectives set beforehand. An example illustrating this is our decision to remove a component targeting anticipated regret from the intervention as it clearly evoked resistance as shown by the focus groups. Based on the feedback from the focus groups (Step 4), we changed the method targeting anticipated regret (Step 3), and pretested the intervention again (Step 4). Thus, we moved back and forth between the steps. Furthermore, not only did we fine-tune the content of the intervention to the mothers' preferences, but also the design of the website was chosen by the mothers. Hence, we adapted the entire intervention to the requirements and preferences of the mothers.

Next to maximizing the likelihood of success, using IM has made the process of intervention development explicit and transparent, providing a road map of the decision-making process and its main outcomes. This will suit the interpretation of strengths and weaknesses of the intervention when looking at the results from the outcome evaluation (Step 6) (74). It also enables the owner to improve the intervention where necessary and others to replicate the steps described when developing a similar intervention for different populations and/or settings (79).

### Design rationales in eHealth

Recently, it has been argued that eHealth researchers should publish descriptions of interventions and results from evaluation studies separately in order to gain a better understanding of what exactly is being evaluated, facilitate comparison between interventions, and extend the evidence base for the development of future interventions (80, 81). The current paper complies to this call and adds to the plea for systematic and detailed descriptions of design rationales in the eHealth field. Systematic descriptions may improve the quality of future systematic reviews that assess the link between design features and outcomes of an intervention (6, 82, 83). These reviews, in turn, can be used as a guide for eHealth researchers in designing future interventions with improved efficacy, reach, and user acceptability (81).

### Limitations

Although we believe that using IM has greatly contributed to the intervention being effective in promoting HPV-vaccination acceptability, we agree with other authors that IM is a complex and time-consuming process (84–86). However, we are convinced that the development of the intervention was brought to a higher level by IM. Moreover, we believe that the experience we gained may improve efficacy of the process and make it less time-consuming in future intervention development. We can profit from this experience when developing similar interventions for a different population (e.g., an intervention promoting HPV-vaccination among Dutch boys).

### Conclusion

In this article, we provide a detailed, comprehensive description of how we systematically developed an intervention promoting HPV-vaccination acceptability. Using IM led to an innovative and effective intervention using interactive Web-based computer-tailored education. This intervention blueprint will aid in interpreting the results of our evaluation studies. Moreover, it will ease comparisons of design rationales across interventions, and may provide leads for the development of other eHealth interventions. Overall, this paper adds to the plea for systematic reporting of design rationales constituting the process of developing interventions, and the development of a cumulative science of interventions in the eHealth field.

## Ethics statement

The study was approved by the Medical Ethical Committee (METC), the ethical committee of the VU Medical Center in Amsterdam. Informed consent was provided online for the online studies (i.e., the three experimental pretests, the online focus group and the RCT). For the focus groups, participants provided written consent.

## Author contributions

MP contributed to study conception and design, acquisition of data, analysis, and interpretation of data, and drafting of manuscript. HvK, TP, and RR contributed to study conception and design, interpretation of data and revising the manuscript critically. AH and HdM contributed to study conception and design and revising the manuscript critically. HvV critically revised the manuscript. All authors approved the final version of the manuscript to be published.

### Conflict of interest statement

The authors declare that the research was conducted in the absence of any commercial or financial relationships that could be construed as a potential conflict of interest.
