# Supplementary material for: Systematically Developing a Web-Based Tailored Intervention Promoting HPV-Vaccination Acceptability Among Mothers of Invited Girls Using Intervention Mapping
Source: Front Public Health. 2018 Sep 28;6:226. doi: 10.3389/fpubh.2018.00226 (PMC6190841; doi:10.3389/fpubh.2018.00226)
Supplement: Supplementary file 1 [file Table_1.DOCX]

**Additional file 1**

Matrices of change objectives

| **Performance Objective** | **Determinant** | | | | |
| --- | --- | --- | --- | --- | --- |
|  | **Knowledge** | **Attitude** | **Beliefs** | **Negative outcome expectancies** | **Positive outcome expectancies** |
| 1. Mother makes the (informed) decision to have her daughter vaccinated against HPV. | Mother explains that HPV is a virus.  Mother explains that HPV is transmitted sexually.  Mother explains HPV is spread by skin-to-skin sexual contact and is prevalent in all sexually active populations.  Mother explains that condoms are ineffective in preventing HPV.  Mother recognizes that her daughter is mandatory to get the vaccination after she has been invited.  Mother recognizes that infection with HPV can persist unnoticed.  Mother explains that men can also be infected with HPV.  Mother recognizes that her daughter can be infected with HPV through sexual contact with a men.  Mother recognizes that it is recommended that her daughter still participates in the national cervical cancer screening program after having received the vaccination.  Mother recognizes that the HPV-vaccination protects against 70% of HPV infections.  Mother describes that the HPV-vaccination includes 2 injections.  Mother recognizes that currently, there are no effective HPV prevention strategies.  Mother recognizes that infection by HPV is the major cause of cervical cancer. | Mother evaluates the HPV-vaccination positively.  Mother recognizes the health benefits of the HPV-vaccination. | Mother recognizes that the vaccine has proven to be safe.  Mother recognizes that the vaccine has proven to be effective.  Mother recognizes that the government shows responsibility for the health of the Dutch population by introducing the HPV-vaccination.  Mother recognizes the importance of her daughter receiving the HPV-vaccination before they become sexually active (i.e., age 12).  Mother recognizes that the HPV-vaccination was introduced for the sake of her daughter and irrespective of the pharmaceutical industry’s interest.    Mother recognizes that the HPV-vaccination is most effective when her daughter gets fully vaccinated.  Mother recognizes that here daughter still has a chance to get infected with HPV, even if she only has one partner.  Mother recognizes that it is still useful to get the HPV-vaccination despite her daughter already having had sex.  Mother recognizes why the HPV-vaccination is given at age 12 (i.e., because it is most effective before they become sexually active). | Mother describes the (potential) negative effects of the HPV-vaccination.  Mother recognizes that if her daughter gets the HPV-vaccination, she might get unpleasant side effects shortly after the injection, such as a painful arm, a red injection spot, crying, fainting, and short-time fatigue.  Mother recognizes that so far, no evidence exists for a relationship between the HPV-vaccination and migraine.  Mother recognizes that no evidence exists between the HPV-vaccination and chronic fatigue.  Mother describes the discrepancy between facts and stories regarding the (potential) negative outcomes of the HPV-vaccination.  Mother recognizes that so far, no evidence exists for a relationship between the HPV-vaccination and the functioning of her daughter’s immune system.  Mother recognizes that so far, no evidence exists for a relationship between the HPV-vaccination and infertility.  Mother recognizes that so far, no evidence exists for a relationship between the HPV-vaccination and her daughter having unsafe sex in the future.  Mother recognizes that so far, no evidence exists for a relationship between the HPV-vaccination and paralysis. | Mother describes the (potential) positive outcomes of the HPV-vaccination.  Mother recognizes that if her daughter gets the HPV-vaccination, she is less likely to contract cervical cancer.  Mother recognizes that if her daughter gets the HPV-vaccination, she will have to worry less about cervical cancer.  Mother recognizes that if her daughter gets the HPV-vaccination, she is less likely to be infected with HPV. |

| **Performance Objective** | **Determinant** | | | | | |
| --- | --- | --- | --- | --- | --- | --- |
|  | **Risk perception having received no HPV-vaccination** | **Risk perception having received the HPV-vaccination** | **Confidence in authorities** | **Subjective norms** | **Descriptive norms** | **Relative effectiveness** |
| 1.Mother makes the (informed) decision to have her daughter vaccinated against HPV. | Mother acknowledges the risk of her daughter becoming infected with HPV and developing cervical cancer later in life without the vaccination. | Mother acknowledges the reduction in risk of her daughter becoming infected with HPV and developing cervical cancer later in life after receiving the vaccination. | Mother has trust in the government’s prevention policies / the Ministry of Public Health.  Mother has trust in science with regards to the HPV-vaccination.  Mother has trust in health care with regards to the HPV-vaccination.  Mother recognizes that the government has implemented the HPV vaccine in the NIP to further reduce the cervical cancer burden in the Netherlands. | Mother knows about the opinion of important others (e.g., her daughter and partner) about the HPV-vaccination.  Mother is able to express resistance to a contrasting opinion about the HPV-vaccination of important others. | Mother recognizes other mothers decide to have their daughter vaccinated against HPV. | Mother describes which factors puts her daughter at risk for developing cervical cancer.  Mother recognizes that the HPV-vaccination is the most effective way to protect against HPV relative to other methods of protection (i.e., having safe sex, having sex with only one person in a lifetime, participating in the cervical cancer screening).  Mother recognizes that no evidence exists for a relationship between having a healthy lifestyle and developing cervical cancer. |

| **Performance Objective** | **Determinant** | | | | |
| --- | --- | --- | --- | --- | --- |
|  | **Anticipated regret rejecting vaccination** | **Anticipated regret receiving vaccination** | **Habit strength** | **HPV-vaccination processing** | **Ambivalence** |
| 1. Mother makes the (informed) decision to have her daughter vaccinated against HPV. | Mother anticipates feelings of regret if her daughter receives no HPV-vaccination and develops cervical cancer later in life.  Mother’s concerns about her daughter getting cervical cancer without the HPV-vaccination outweigh concerns about her daughter contracting a serious illness because of the HPV-vaccination.  Mother anticipates feelings of regret if her daughter is only partially vaccinated and develops cervical cancer later in life. | Mother’s concerns about her daughter getting cervical cancer without the HPV-vaccination outweigh concerns about her daughter contracting a serious illness because of the HPV-vaccination. | Mother is motivated to think about the HPV-vaccination | Mother actively processes information about the HPV-vaccination. | Mother’s ambivalence towards the HPV-vaccination decision is resolved.  Mother experiences more positive than negative feelings during their decision making about her daughter’s HPV-vaccination.  Mother ambivalence between feelings and cognitions of the vaccination is resolved.  Mother recognizes more pros than cons of the HPV-vaccination. |

| **Performance Objective** | **Determinant** | |
| --- | --- | --- |
|  | **Attitude** | **Self-efficacy** |
| 2. Mother discusses her decision to have her daughter vaccinated against HPV with her daughter and important others. | Mother evaluates communication with important others positively. | Mother expresses confidence in discussing the decision to have her daughter vaccinated against HPV with important others. |

| **Performance Objective** | **Determinant** | |
| --- | --- | --- |
|  | **Knowledge** | **Beliefs** |
| 3. Mother guides her daughter towards receiving the first HPV-injection.  4. Mother guides her daughter towards receiving both HPV-injections. | Mother knows where to get the first HPV-injection.  Mother describes that the second HPV-injection has to be received 6 months after the first HPV-injection.  Mother knows where to get the second HPV-injection. | Mother recognizes that the HPV-vaccination is most effective when her daughter gets fully vaccinated. |
