# Supplementary material for: Systematically Developing a Web-Based Tailored Intervention Promoting HPV-Vaccination Acceptability Among Mothers of Invited Girls Using Intervention Mapping
Source: Front Public Health. 2018 Sep 28;6:226. doi: 10.3389/fpubh.2018.00226 (PMC6190841; doi:10.3389/fpubh.2018.00226)
Supplement: Supplementary file 2 [file Table_2.DOCX]

**Additional file 2**

Intervention blueprint: components, performance objectives (POs), determinants, change objectives (COs), methods and strategies.

| **Menu** | **Component** | **PO** | **Determinant*** | **Change Objectives** | **Methods** | **Applications** |
| --- | --- | --- | --- | --- | --- | --- |
| Throughout the entire intervention | All components | 1^a^ | HPV-vaccination processing  Habit strength | Mother actively processes information about the HPV-vaccination.  Mother is motivated to think about the HPV-vaccination. | All of the methods described below. | All of the strategies described below. |
| Information about the HPV-vaccination | General information | 1 | Knowledge  Risk perception having received the HPV-vaccination | Mother explains that HPV is a virus.  Mother explains that HPV is transmitted sexually.  Mother explains HPV is spread by skin-to-skin sexual contact and is prevalent in all sexually active populations.  Mother explains that condoms are ineffective in preventing HPV.  Mother recognizes that her daughter is not mandatory to get the vaccination after she has been invited.  Mother recognizes that infection with HPV can persist unnoticed.  Mother explains that men can also be infected with HPV.  Mother recognizes that her daughter can be infected with HPV through sexual contact with a men.  Mother recognizes that it is recommended that her daughter still participates in the national cervical cancer screening program after having received the vaccination.  Mother recognizes that the HPV-vaccination protects against 70% of HPV infections.  Mother describes that the HPV-vaccination includes 2 injections.  Mother recognizes that currently, there are no effective HPV prevention strategies.  Mother describes that the second HPV-injection has to be received 6 months after the first HPV-injection.  Mother acknowledges the reduction in risk of her daughter becoming infected with HPV and developing cervical cancer later in life after receiving the vaccination. | Feedback (Theories of Learning) | Mothers are provided with basic information about HPV, cervical cancer, and the HPV-vaccination. |
|  | Facts and stories | 1 | Beliefs  Negative outcome expectancies  Confidence in authorities  Knowledge | Mother recognizes that her daughter still has a chance to get infected with HPV, even if she only has one partner.  Mother recognizes that the vaccine has proven to be safe.  Mother recognizes that the vaccine has proven to be effective.  Mother recognizes that the government shows responsibility for the health of the Dutch population by introducing the HPV-vaccination.  Mother recognizes that it is still useful to get the HPV-vaccination despite her daughter already having had sex.  Mother recognizes that the HPV-vaccination was introduced for the sake of her daughter and irrespective of the pharmaceutical industry’s interest  Mother recognizes that so far, no evidence exists for a relationship between the HPV-vaccination and the functioning of her daughter’s immune system.  Mother recognizes that so far, no evidence exists for a relationship between the HPV-vaccination and her daughter having unsafe sex in the future.  Mother recognizes that so far, no evidence exists for a relationship between the HPV-vaccination and infertility.  Mother describes the discrepancy between facts and stories regarding the (potential) negative outcomes of the HPV-vaccination.  Mother has trust in the government’s prevention policies / the Ministry of Public Health.  Mother has trust in science with regards to the HPV-vaccination.  Mother has trust in health care with regards to the HPV-vaccination.  Mother recognizes that the government has implemented the HPV vaccine in the NIP to further reduce the cervical cancer burden in the Netherlands.  Mother recognizes that infection by HPV is the major cause of cervical cancer.  Mother explains HPV is spread by skin-to-skin sexual contact and is prevalent in all sexually active populations.  Mother recognizes that it is recommended that her daughter still participates in the national cervical cancer screening program after having received the vaccination. | Active learning (Social Cognitive Theory, Elaboration Likelihood Model)  Feedback (Theories of Learning) | Mothers are asked by the mother-like assistant to indicate for various statements (e.g., ‘If my daughter gets vaccinated against HPV, she might become infertile’), whether they are either a ‘fact’ (true) or a ‘story’ (false).Then, the doctor-like virtual assistant elaborates on correct outcome expectancies, beliefs, misperceptions and omissions. After she has provided the tailored feedback, additional written information about the topic appears.  Also, mothers are given the opportunity to read more about the implementation of the HPV-vaccination in the NIP (e.g., about the role of the government) and watch a video about this topic. |
|  | From HPV to cervical cancer | 1 | Knowledge | Mother recognizes that infection by HPV is the major cause of cervical cancer. | Active learning (Social Cognitive Theory, Elaboration Likelihood Model) | Mothers are provided with a video showing how an infection with HPV could lead to cervical cancer. |
|  | Ways to protect against cervical cancer | 1 | Relative effectiveness  Knowledge  Beliefs  Risk perception having received the HPV-vaccination  Risk perception having received no HPV-vaccination | Mother describes which factors puts her daughter at risk for developing cervical cancer.  Mother recognizes that the HPV-vaccination is the most effective way to protect against HPV relative to other methods of protection (i.e., having safe sex, having sex with only one person in a lifetime, participating in the cervical cancer screening).  Mother recognizes that no evidence exists for a relationship between having a healthy lifestyle and developing cervical cancer.  Mother explains that condoms are ineffective in preventing HPV.  Mother explains that HPV is transmitted sexually.  Mother explains HPV is spread by skin-to-skin sexual contact and is prevalent in all sexually active populations.  Mother recognizes that it is recommended that her daughter still participates in the national cervical cancer screening program after having received the vaccination.  Mother recognizes that her daughter still has a chance to get infected with HPV, even if she only has one partner.  Mother recognizes that her daughter is likely to be serial monogamous rather than monogamous (unrealistic optimism).  Mother recognizes that the vaccine has proven to be effective.  Mother acknowledges the reduction in risk of her daughter becoming infected with HPV and developing cervical cancer later in life after receiving the vaccination.  Mother acknowledges the risk of her daughter becoming infected with HPV and developing cervical cancer later in life without the vaccination. | Advance organizers (Theories of Information Processing)  Active learning (Social Cognitive Theory, Elaboration Likelihood Model)  Feedback (Theories of Learning) | Mothers are asked by the mother-like assistant how effective she considers alternative methods of protecting against cervical cancer (i.e., having safe sex, having sex with only one person in a lifetime, participating in the cervical cancer screening) to be. Then, the doctor-like assistant provides tailored feedback. After the assistant has provided the feedback, additional written information about the specific topic appears. |
|  | Side effects of the HPV-vaccination | 1 | Negative outcome expectancies | Mother describes the (potential) negative effects of the HPV-vaccination.  Mother recognizes that if her daughter gets the HPV-vaccination, she might get unpleasant side effects shortly after the injection, such as painful arm, a red injection spot, crying, fainting, and short-term fatigue  Mother recognizes that no evidence exists for a relationship between the HPV-vaccination and migraine.  Mother recognizes that no evidence exists between the HPV-vaccination and chronic fatigue.  Mother describes the discrepancy between facts and stories regarding the (potential) negative outcomes of the HPV-vaccination  Mother recognizes that so far, no evidence exists for a relationship between the HPV-vaccination and the functioning of her daughter’s immune system.  Mother recognizes that so far, no evidence exists for a relationship between the HPV-vaccination and infertility.  Mother recognizes that so far, no evidence exists for a relationship between the HPV-vaccination and paralysis. | Belief selection (Theory of Reasoned Action)  Feedback (Theories of Learning)  Active learning (Social Cognitive Theory, Elaboration Likelihood Model) | First, the mother-like assistant provides a list including potential short-term side effects (i.e., painful arm, a red injection spot, crying, fainting, and short-term fatigue). Mothers are asked to indicate whether evidence exists or not per potential side effect. Then, the doctor-like assistant provides tailored feedback. After the assistant has provided the feedback, additional written information about the specific topic appears Then, mothers are presented with another list by the mother-like virtual assistant, this time including potential long-term side effects (i.e., migraine, chronic fatigue, paralysis, deficits in immune system, and infertility). Again, she is asked to indicate whether prove exists per potential long-term side effect. Then, the doctor-like assistant provides tailored feedback. After the assistant has provided the feedback, additional written information about the specific topic appears |
|  | Importance vaccinating at young age | 1 | Beliefs | Mother recognizes why the HPV-vaccination is given at age 12 (i.e., because it is most effective before they become sexually active).  Mother recognizes the importance of her daughter receiving the HPV-vaccination before they become sexually active (i.e., age 12).  Mother recognizes that it is still useful to get the HPV-vaccination despite her daughter already having had sex. | Belief selection (Theory of Reasoned Action)  Feedback (Theories of Learning)  Active learning (Social Cognitive Theory, Elaboration Likelihood Model) | The mother-like virtual assistant asks the mothers to indicate whether she agrees/disagrees/is neutral with the following statement: ‘My daughter is too young to receive the HPV-vaccination. Then, the doctor-like assistant provides tailored feedback. After the assistant has provided the feedback, additional written information about the specific topic appears. The same was done for the following statements: ‘my daughter is not yet sexually active, so she does not need the HPV-vaccination’ or (if mother indicates her daughter is already sexually active) ‘my daughter is already sexually active, so it is not useful anymore to get her vaccinated against HPV-vaccination.’ |
|  | Other mothers | 1 | Descriptive norms | Mother recognizes other mothers decide to have their daughter vaccinated against HPV. | Modeling (Social Cognitive Theory) | Mothers are first asked about what they think mothers in their environment do (vaccinate their daughter against HPV / don’t vaccinate their daughter against HPV / don’t know). Then, they are provided with tailored feedback and information on the national HPV-vaccination uptake. |
|  | Working mechanisms vaccination | 1 | Knowledge | Mother recognizes that infection by HPV is the major cause of cervical cancer.  Mother describes that the HPV-vaccination includes 2 injections.  Mother describes that the second HPV-injection has to be received 6 months after the first HPV-injection. | Active learning (Social Cognitive Theory, Elaboration Likelihood Model) | Mothers are provided with a video showing how the HPV-vaccination works to protect her daughter against infection with HPV. |
|  | Chance of getting HPV/  cervical cancer | 1 | Risk perception having received (no) HPV-vaccination | Mother acknowledges the risk of her daughter becoming infected with HPV and developing cervical cancer later in life without the vaccination.  Mother acknowledges the reduction in risk of her daughter becoming infected with HPV and developing cervical cancer later in life after receiving the vaccination. | Statistical risk information (Health Belief Model)  Consciousness raising (Health Belief Model)  Framing (Protection Motivation Theory) | Mother-like assistant asks about mothers’ perceived risk perception of her daughter getting infected with HPV and of her daughter developing cervical cancer. Tailored feedback on this perceived risk is then given by the doctor-like assistant. Finally, after the doctor mothers are provided with statistical risk information (i.e., the probability rates of attracting HPV and cervical cancer). |
|  | Effectiveness and safety of the HPV-vaccination | 1 | Positive outcome expectancies  Beliefs | Mother describes the (potential) positive outcomes of the HPV-vaccination.  Mother recognizes that if her daughter gets the HPV-vaccination, she is less likely to contract cervical cancer.  Mother recognizes that if her daughter gets the HPV-vaccination, she will have to worry less about cervical cancer.  Mother recognizes that if her daughter gets the HPV-vaccination, she is less likely to be infected with HPV.  Mother recognizes that the HPV-vaccination effectively protects against cervical cancer.  Mother recognizes that the HPV-vaccination also protects against other types of cancer (e.g., anal cancer).  Mother recognizes that the HPV-vaccination is most effective when her daughter gets fully vaccinated. | Belief selection (Theory of Reasoned Action)  Feedback (Theories of Learning)  Active learning (Social Cognitive Theory, Elaboration Likelihood Model) | Mothers are asked for her opinion by the mother-like assistant about the following 2 statements regarding the effectiveness of the HPV-vaccination (disagree / don’t know / agree): 1) if my daughter gets vaccinated against HPV, she will not get infected with HPV, and 2) if my daughter gets vaccinated against HPV, she will not develop cervical cancer. Then, the doctor-like assistant provides tailored feedback. After the assistant has provided the feedback, additional written information about the specific topic appears. |
| Weighing up the pros and cons | Decisional Balance | 1 | Attitude,  Ambivalence | Mother evaluates the HPV-vaccination positively.  Mother recognizes the health benefits of the HPV-vaccination.  Mother’s ambivalence towards the HPV-vaccination decision is resolved.  Mother experiences more positive than negative feelings during their decision making about her daughter’s HPV-vaccination.  Mother’s ambivalence between feelings and cognitions of the vaccination is resolved.  Mother recognizes more pros than cons of the HPV-vaccination. | Decisional Balance (Motivational Interviewing) | Mothers are presented with a list of pros and cons of the HPV-vaccination by the mother-like assistant. Based on pros and cons mothers marked as most salient, a decisional balance reveals their current position on a scale ranging between wanting and not-wanting to get my daughter vaccinated. |
|  | Values clarification | 1 | Attitude  Ambivalence | Mother evaluates the HPV-vaccination positively.  Mother recognizes the health benefits of the HPV-vaccination.  Mother’s ambivalence towards the HPV-vaccination decision is resolved. | Value Clarification (Motivational Interviewing)  Modeling (Social Cognitive Theory) | Mothers are invited to list their central values for life. Optional, they can find examples of values of other mothers (e.g., being a good parent). They will then be stimulated to relate these to the HPV-vaccination. Here, examples of how these values were related to the HPV-vaccination according to other mothers, were available. |
| Practical information | Talking about the HPV-vaccination | 1, 2^b^ | Subjective norms  Attitude  Self-efficacy | Mother knows about the opinion of important others (e.g., her daughter and partner) about the HPV-vaccination.  Mother is able to express resistance to a contrasting opinion about the HPV-vaccination of important others.  Mother evaluates communication with important others positively.  Mother expresses confidence in discussing the decision to have her daughter vaccinated against HPV with important others. | Feedback (Theories of Learning)  Resistance to social pressure (Reasoned Action Approach)  Modeling (Social Cognitive Theory)  Information about others’ approval (Reasoned Action Approach) | Mothers are asked to indicate with whom they want to discuss the HPV-vaccination from a list: with their daughter, partner, general practitioner, other family members, and/or other parents. When the daughter was not chosen from this list, the importance of discussing the HPV-vaccination with their daughter is emphasized by the mother-like virtual assistant. Then, mothers are asked if they expect difficulties discussing the HPV-vaccination with their important others. The mother-like assistants then provides tailored feedback and advice on how to discuss the HPV-vaccination. |
|  | Where do I get the HPV-vaccination | 3^c^, 4^d^ | Knowledge | Mother knows where to get the first HPV-injection.  Mother knows where to get the second HPV-injection. |  | Mothers are provided with information about where and to get the HPV-vaccination. |
|  | 2 instead of 3 HPV-injections: |  | Knowledge | Mother describes that the HPV-vaccination includes 2 injections. |  | Mothers are provided with information about why their daughter only needs 2 instead of previously 3 HPV-injections. |
| Frequently asked questions | Frequently asked questions about the HPV-vaccination | n/a^e^ | n/a | n/a | n/a | n/a |
|  | Frequently asked questions about getting the HPV-vaccination: knowledge | n/a | n/a | n/a | n/a | n/a |
|  | Problems with the website | n/a | n/a | n/a | n/a | n/a |

^a^ PO1: Mother makes the (informed) decision to have her daughter vaccinated against HPV.

^b^ PO2: Mother discusses her decision to have her daughter vaccinated against HPV with her daughter and important others.

^c^ PO3: Mother guides her daughter towards receiving the first HPV-injection.

^d^ PO4: Mother guides her daughter towards receiving the second HPV-injection.

^e^ n/a = not applicable

* Determinants are ordered based on their importance; the first determinant in line is the main determinant targeted.
