# Supplementary material for: Systematically Developing a Web-Based Tailored Intervention Promoting HPV-Vaccination Acceptability Among Mothers of Invited Girls Using Intervention Mapping
Source: Front Public Health. 2018 Sep 28;6:226. doi: 10.3389/fpubh.2018.00226 (PMC6190841; doi:10.3389/fpubh.2018.00226)

Additional file 3

Screenshots of the four menus of the website

Picture 1: The first menu of the website ('information about the HPV-vaccination').

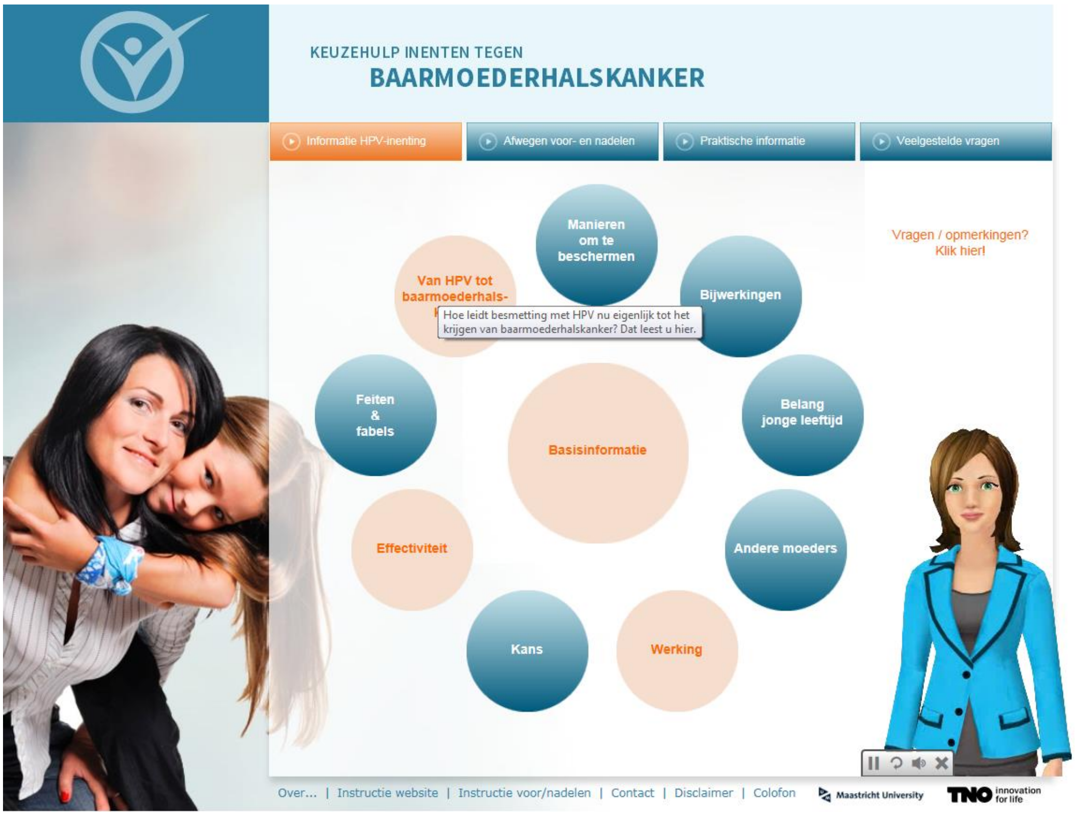

Picture 2: The second menu of the website ('weighing up the pros against the cons').

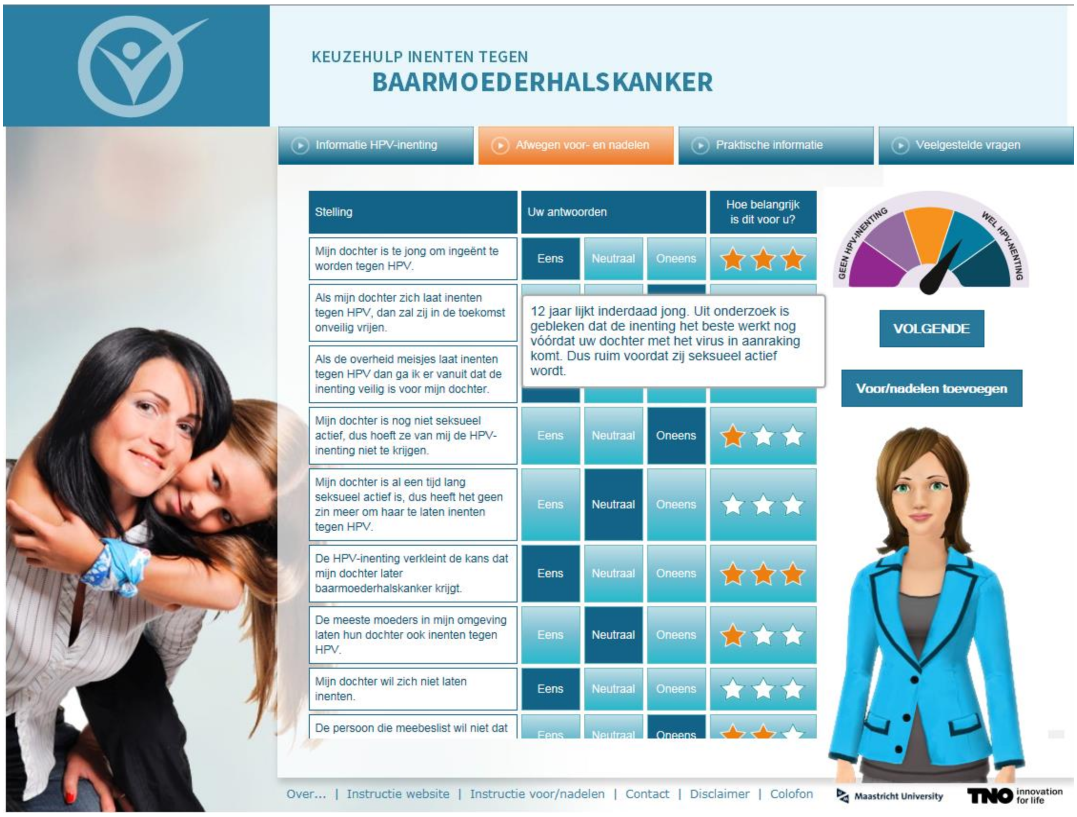

Picture 3: The third menu of the website ('practical information').

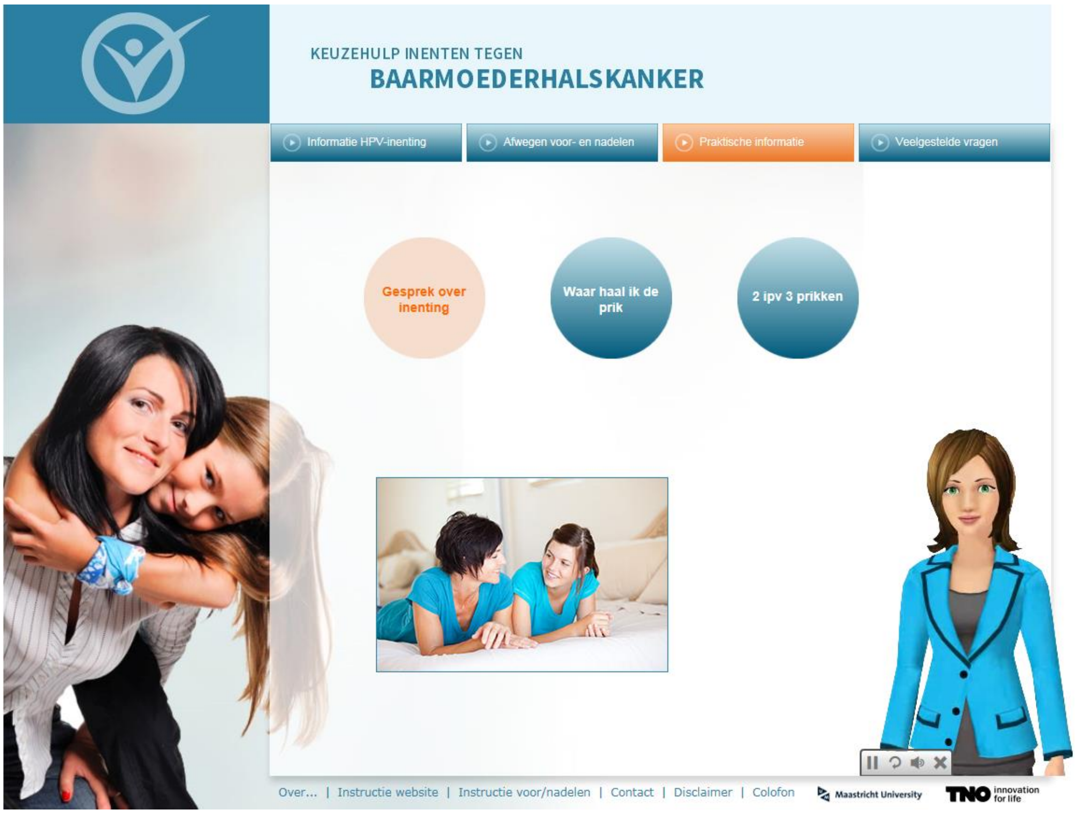

Picture 4: The fourth menu of the website ('frequently asked questions').

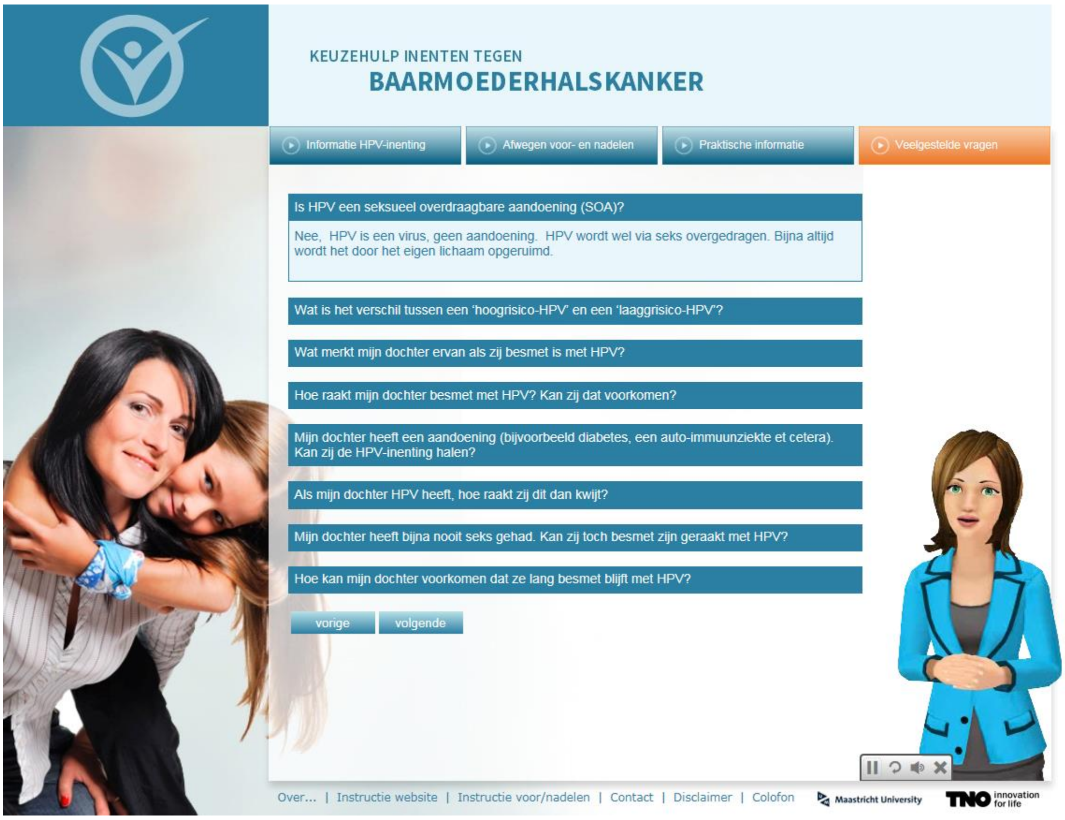

Supplement: Supplementary file 3 [file Presentation_1.PDF]
